# Article Processing Charge Waiver Policies as a Barrier to Oncology Scholarship in Low- and Lower-Middle-Income Countries

**DOI:** 10.1200/GO.21.00143

**Published:** 2021-09-23

**Authors:** Ulysses G. Gardner, Petria S. Thompson, Jason Burton, Caleb Stewart, C. David Fuller, Michael K. Rooney, Ethan B. Ludmir

**Affiliations:** ^1^ Kettering Health, Dayton, OH; ^2^ St Mary's Medical Center, San Francisco, CA; ^3^ Dartmouth-Hitchcock Medical Center, Lebanon, NH; ^4^ Unity Health, Searcy, AR; ^5^ The University of Texas MD Anderson Cancer Center, Houston, TX

Open access (OA) publication aims to increase transparency and reproducibility in scientific research, thereby providing equal access to discovery and knowledge.^1^ Moreover, OA publishing allows innovative research to reach a global audience without additional cost to consumers.^2^ The importance of accessibility through OA has been made particularly evident in the worldwide rapid sharing of research and data during the COVID-19 pandemic, as medical and scientific communities collaborate to develop preventive and therapeutic strategies for the novel severe acute respiratory syndrome coronavirus 2 (SARS-CoV-2).^3^ However, OA publishing in scientific journals is often associated with significant article processing charges (APCs) that may hinder publication; in particular, the financial barrier posed by APCs may disproportionately affect scholarship from low- and lower-middle-income countries (LMICs).

APC waivers for LMIC authors may help to address this barrier. Generally, APC waiver eligibility is determined by the specific journal or publisher, using either the gross national income (GNI) per capita or the gross domestic product of a given country.^4,5^ Gross domestic product tends to reflect a nation's overall population size, whereas GNI per capita reflects the per capita income; the World Bank classifies countries based on GNI per capita into low-, low-middle, upper-middle, and high-income groups.^4,6^ The World Bank classification system is generally used to determine LMIC status for the purposes of APC waivers, although some journal- and publisher-specific variation exists.

In this investigation, we sought to evaluate the landscape of OA publishing in oncology through an observational study of global oncology journal publishing practices, with a particular emphasis on individual journal policies regarding APC waivers for authors from LMICs. We therefore characterized the incidence and factors associated with APC waivers for LMIC authors. Through absence of LMIC APC waivers, we propose that journals and publishers may be creating unnecessary barriers toward a shared mission of open global scientific advancement.

We analyzed a major journals database to assess LMIC APC waiver policies over a large collection of journals. To that end, the SCImago Journal & Country Rank database was queried on August 19, 2020, to identify oncology journals.^7^ SCImago Journal & Country Rank is a publicly available portal, using information from Scopus (Elsevier B.V.), which ranks scholarly journals based on the number of citations received over the previous three years and the relative prestige of the journal.^7,8^ Resulting journals were screened according to OA publishing status. Journals with an OA publishing option (hybrid or full) and APC data available via their website were included. Three-hundred sixty-seven journals were identified by initial search results, of which 272 met inclusion criteria for analysis. Hybrid OA refers to subscription-based journals that allow authors an option of making individual articles OA, and therefore immediately available to the public, following payment of an APC. Full OA journals, by contrast, are those in which all articles are OA and made publicly available upon payment of the APC.^9^ Journals that were discontinued or written in non-English language were excluded for this analysis. For all included journals in this analysis, journal and published websites were manually searched with extraction of data regarding: OA type (hybrid or full), APC amount (US dollars [USD]), presence of an APC waiver for LMIC authors, continent of origin, primary treatment modality (radiation, surgical, medical, or unspecified or general oncology), disease-site-specificity (yes or no), and SCImago journal impact quartile. Regarding LMIC waivers, no distinction was made between waivers for authors from low- versus lower-middle-income countries. Any waiver based on lower economic status of country-of-origin was counted as an LMIC waiver for purposes of the present analysis.

Of 272 journals, 51.5% (140 of 272) offered an APC waiver to authors from LMICs (Table 1). The median APC for all journals was 2,810 (0-5,200) USD; however, journals offering an LMIC waiver had lower APCs than those not offering waivers (median 2,490 *v* 3,260 USD, respectively, *P* < .001). The average APC for quartile 1 (Q1) journals was 3,285 USD versus 2,714 USD, 2,001 USD, and 1,356 USD for Q2, Q3, and Q4 journals, respectively.

**TABLE 1:**
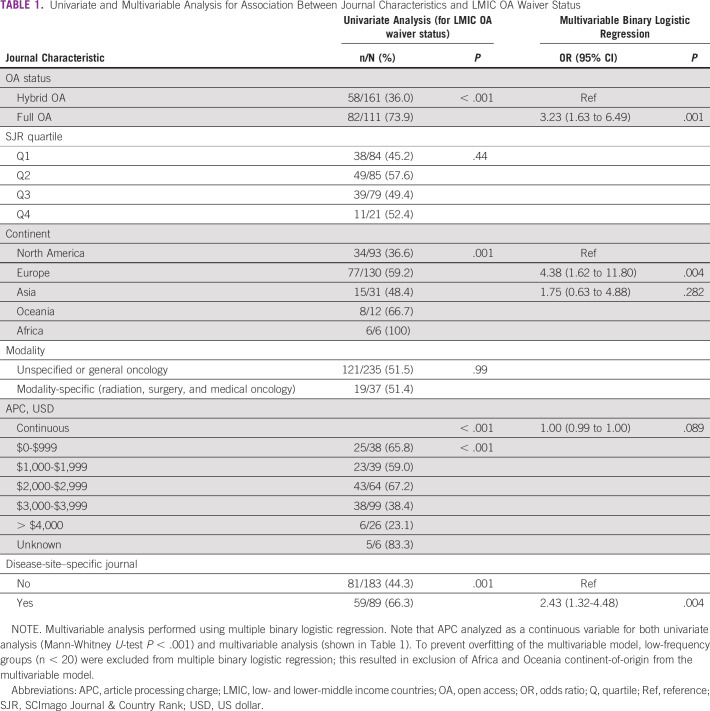
Univariate and Multivariable Analysis for Association Between Journal Characteristics and LMIC OA Waiver Status

On univariate analysis, journals that were full OA (compared with hybrid OA), disease-site-specific, and those with lower APCs were more likely to offer APC waivers to authors from LMICs (Table 1). Journals based in North America were least likely to offer APC waivers to LMIC authors, particularly compared with Europe-based journals (Table 1). Journal impact quartile and modality-specific status were not associated with LMIC waiver status. Multivariable analysis was then performed using multiple binary logistic regression to identify factors independently associated with LMIC APC waiver status. On multivariable analysis, journal full OA status (*P* = .001), Europe-based journals (*P* = .004), and disease-site-specificity (*P* = .004) all were independently associated with LMIC waiver status, with a trend toward increased APC being associated with lower likelihood of LMIC waiver (*P* = .089; Table 1).

In this comprehensive evaluation of oncology journal OA publishing practices, we found that journals with hybrid OA status, higher APCs, and those from North America were seemingly less likely to offer APC waivers to LMIC authors. These findings suggest that inherent structural barriers exist which may limit the ability of scholars from LMICs to equitably share scientific research across global platforms. Particularly in light of an estimated 81% increase in cancer incidence in LMICs expected over the next two decades,^10^ ensuring effective distribution of global oncology research is imperative. Mitigating financial barriers to dissemination of global oncology research represents an important component in achieving this objective.

Open science represents an impressive opportunity to effectively disseminate academic research to global audiences. There has been rampant growth in OA journal article publishing over the past three decades, with estimates of a 30% yearly increase in OA articles since 2000.^11^ By removing barriers to access such as article purchasing or journal subscription fees, the OA publishing model theoretically could increase the outreach of research and encourage scientific progress and collaboration. Many observational studies have indeed shown that OA publishing is associated with greater scientific impact according to traditional bibliometrics (such as citation rates).^12-16^ Although it is unclear whether such relationships are causative in nature (or are primarily driven by confounding factors), OA publishing has consistently been shown to increase the visibility of scientific articles, an effect independently observed even in the setting of randomized studies.^17,18^ Therefore, it is paramount that efforts are made to provide fair and equitable opportunity for authors to pursue OA publishing in order for all scholars to benefit from open science.

Authors from LMIC may be disadvantaged as participants in the global oncology OA platform. Although consumers benefit from the removal of all financial barriers to access, costs may now be shifted to submitting authors, who often face high APCs to publish their work. Such charges may act as a significant barrier for researchers considering OA article submission and would only be expected to be even more limiting for authors from LMIC.^19^ Fortunately, many academic and journal organizations have developed strategies to overcome these financial hurdles. For example, some institutions have created OA funding pools to distribute to investigators to aid with submissions. Furthermore, as analyzed here, many journals have implemented policies to offer APC waivers specifically for authors from LMICs. Such policies are crucial for encouraging realistic opportunities for LMIC researchers to optimally share their work. Unfortunately, as we have demonstrated, significant variation exists in APC waiver policies among oncology journals, which may have the consequence of introducing or exacerbating disparities in global oncologic research.

Hybrid OA journals or publishers are uniquely positioned to receive substantially increased revenue by charging publishing fees and subscription charges for access to journal articles. This may lead to authors and institutions paying twice for access to publications, and leads to concern of these publishers double-dipping in already-limited institutional resource funding pools. In our analysis, the average cost of publishing charges was significantly higher in hybrid OA journals as compared to their full OA counterparts (3,161 *v* 1,671 USD, respectively). Multivariable analysis confirmed that hybrid journals in our cohort were less likely to offer an APC waiver for LMIC authors. These data should encourage publishers adopting the hybrid OA model to consider implementing an LMIC waiver to ensure fairness in the sharing and reproducibility of data.

Journals with the highest APCs also appear to offer waivers less frequently than more affordable journals. Moreover, journals with the most costly publication charges tended to be those with higher impact factors; in our analysis, 71.1% (59 of 83) of the highest-impact-quartile journals charged APCs ≥ 3,000 USD, with a significant association between APC and impact quartile (*P* < .001). Because high-impact journals are often highly regarded and sought-after by researchers as potential publication forums, these results suggest that authors from LMICs may be further disadvantaged when attempting to publish research in highly visible and respected forums. This observed trend of journals with high-APC high-impact journals being less likely to offer LMIC waivers is troubling, and may lead to a loss of high-impact science from global colleagues.

Geographic differences in LMIC APC waiver status are also noted, with North American journals among the least likely to offer LMIC authors APC waivers. Journals based in North America are among those with the highest impact (38.7% of North American journals are in the highest-impact quartile, the highest proportion for any continent [*P* < .001]) and highest APC charges (58.2% of North American journals charge APC ≥ 3,000 USD, the highest of any region [*P* < .001]). With higher costs, higher impact, and fewer LMIC waivers, North American journals and publishers should reconsider their LMIC waiver policies to optimize visibility and representation of global oncology research. Additionally, disease-site–specific (ie, breast, lung, and prostate) journals were more likely to offer LMIC APC waivers compared with more general non–site-specific publications. Although the reasons for this observation are likely multifactorial, one consideration may be that disease-site–specific journals are often linked to disease-site–specific societies and organizations, which may be more willing to offer LMIC waivers to promote scholarship from across the globe in a given disease site. Society-level policies with the express goal of promoting global research may contribute to this finding as well.

As with any cross-sectional observational study, our investigation has methodologic limitations. First, although we used a systematic approach for identifying potential oncology journals via the SCImago database, it is possible that some journals that would have otherwise met inclusion criteria were not listed in this database and therefore were not included. Second, we limited our analysis to English-language only, which may introduce bias and limit the generalizability of our findings to non-English research platforms, particularly in the context of global oncology literature. Finally, this analysis relied upon public disclosure by journals of both LMIC APC waiver policy as well as APC fees themselves. Some 78 journals were excluded from this analysis owing to no publicly available data regarding APC fees. It is conceivable that among both included and excluded journals, journals may have APC waiver policies not publicly available that were not captured in this study. These nonpublic APC waiver policies may directly refer to authors from LMICs, or else provide dispensation for authors with limited resources who request an APC waiver (and thus potentially impact authors from LMICs without explicit mention of LMIC status). Although our study is limited by only relying upon publicly available data, journals with LMIC APC waivers should be encouraged to post such policies publicly, further normalizing the practice of LMIC APC waivers as well as incentivizing submissions by LMIC authors.

Collectively, LMIC APC waiver policies can help foster scholarship and scientific visibility in facilitating OA publication. With only half of analyzed journals offering LMIC APC waivers, and particularly low rates of waivers available for high-cost and hybrid-OA journals, work is still needed to promote equity in the scientific publishing process.
